# Termite mound cover and abundance respond to herbivore‐mediated biotic changes in a Kenyan savanna

**DOI:** 10.1002/ece3.7445

**Published:** 2021-06-03

**Authors:** Grace K. Charles, Corinna Riginos, Kari E. Veblen, Duncan M. Kimuyu, Truman P. Young

**Affiliations:** ^1^ Department of Plant Sciences University of California Davis California USA; ^2^ Mpala Research Centre Nanyuki Kenya; ^3^ The Nature Conservancy Lander Wyoming USA; ^4^ Department of Wildland Resources and Ecology Center Utah State University Logan Utah USA; ^5^ Department of Natural Resource Management Karatina University Karatina Kenya

**Keywords:** ecosystem engineers, exclosure experiments, large mammalian herbivores, livestock, tree thinning

## Abstract

Both termites and large mammalian herbivores (LMH) are savanna ecosystem engineers that have profound impacts on ecosystem structure and function. Both of these savanna engineers modulate many common and shared dietary resources such as woody and herbaceous plant biomass, yet few studies have addressed how they impact one another. In particular, it is unclear how herbivores may influence the abundance of long‐lived termite mounds via changes in termite dietary resources such as woody and herbaceous biomass. While it has long been assumed that abundance and areal cover of termite mounds in the landscape remain relatively stable, most data are observational, and few experiments have tested how termite mound patterns may respond to biotic factors such as changes in large herbivore communities. Here, we use a broad tree density gradient and two landscape‐scale experimental manipulations—the first a multi‐guild large herbivore exclosure experiment (20 years after establishment) and the second a tree removal experiment (8 years after establishment)—to demonstrate that patterns in *Odontotermes* termite mound abundance and cover are unexpectedly dynamic. Termite mound abundance, but areal cover not significantly, is positively associated with experimentally controlled presence of cattle, but not wild mesoherbivores (15–1,000 kg) or megaherbivores (elephants and giraffes). Herbaceous productivity and tree density, termite dietary resources that are significantly affected by different LMH treatments, are both positive predictors of termite mound abundance. Experimental reductions of tree densities are associated with lower abundances of termite mounds. These results reveal a richly interacting web of relationships among multiple savanna ecosystem engineers and suggest that termite mound abundance and areal cover are intimately tied to herbivore‐driven resource availability.

## Graphical abstract

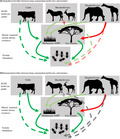

## 1 INTRODUCTION

Understanding the causes and controls of community heterogeneity and structure has long been a goal of ecologists and can provide insight into broader ecosystem dynamics (Lundholm, 2009; Stevens & Tello, 2011; Tews et al., 2004). Ecosystem engineers, organisms that create and modify habitats, are widespread worldwide and can drive ecological and evolutionary patterns (Coggan et al., 2018; Jones et al., 1994, 1997; Wright & Jones, 2006). Ecosystem engineers can create “patches” of contrasting habitats (e.g., beaver dams, coral reefs, salt marshes, termite mounds, wallows, and grazing lawns) that are important sources of habitat heterogeneity (Boogert et al., 2006; Crooks, 2002). Some patch types may persist over time periods ranging from years to millennia (Hastings et al., 2007; Jones et al., 1997; Romero et al., 2014). The effects of ecosystem engineers on landscape heterogeneity depend on their interactions and feedbacks with biotic and abiotic environmental drivers (Romero et al., 2014; Wright et al., 2004).

A number of studies have theoretically explored the dynamics of the abundance, longevity, and stability of engineered patches (Cuddington et al., 2009; Gurney & Lawton, 1996; Hastings et al., 2007; Wright et al., 2004). However, these dynamics have rarely been tested experimentally (but see Lagendijk et al., 2016; Porensky & Veblen, 2015). Models predict that the proportion of landscape occupied as engineered patches versus background habitat depends on the population size, density‐dependent feedbacks, and behavior of ecosystem engineers (Cuddington et al., 2009; Wright, 2009; Wright et al., 2004). Changes in abiotic or biotic conditions may alter ecosystem engineer resource availability and population dynamics, therefore, changing the proportion of engineered landscape. For example, an increase in dietary resources could lead to a population increase of ecosystem engineers and, subsequently, the proportion of engineered landscape.

One globally distributed type of ecosystem engineer, termites, create mounds that generate landscape heterogeneity by locally enriching soil (Jones, 1990), altering biogeochemical cycles (Fox‐Dobbs et al., 2010), changing hydrology and soil structure (Mando, 1997; Mando et al., 1996), and shifting plant palatability and community structure (Okullo & Moe, 2012). Termite mounds can positively influence the productivity, biodiversity, and resilience of savanna ecosystems (Bonachela et al., 2015; Joseph et al., 2014; Okullo & Moe, 2012; Pringle et al., 2010). Fungus‐cultivating termites are known to consume a wide diet that includes live and dead wood and herbaceous material, as well as animal byproducts such as dung and hooves (Freymann et al., ,^2007^, ^2008^; Wood, 1978). Understanding the drivers of termite mound abundance can provide important insight into patterns in savanna ecosystem heterogeneity and biodiversity.

Recent work has shown that termite mound abundance and distribution both in space and time may be modulated by variation in abiotic and biotic resources. Termite mound communities can vary with changes in soil type, rainfall, ecosystem productivity, and herbivore presence (Davies et al., 2014; Haverty et al., 1975; Korb & Linsenmair, 2001), and termite mounds appear to increase in density with availability of dietary resources such as tree density (Davies et al., 2014; Levick et al., 2010), and decrease with intensive human land use (Davies et al., 2020). Some evidence also suggests that termite diets may shift in response to changing availability of plant resources (Boutton et al., 1983; Lepage et al., 1993).

Mature (larger) termite mounds are often regularly spaced (over‐dispersed) in the landscape (Davies et al., 2014; Grohmann et al., 2010; Pringle et al., 2010; Sileshi et al., 2010); these regular‐spaced arrangements of mature mounds have led some to infer that termite mound abundance and the proportion of engineered habitat are relatively stable in space and time, apparently over time spans of many years, and multiple termite generations (Pringle & Tarnita, 2017). However, the temporal stability of these patterns is not well understood. On the one hand, some termite mounds in Africa have been found to be hundreds to thousands of years old (Erens et al., 2015) and studies have reported landscape‐scale stability in mound abundance over multi‐year periods (Pomeroy, 2005). On the other hand, some studies have observed that termite activity, abundance, and diversity can change in response to habitat disturbance over decadal time scales (Eggleton et al., 2002; Jones et al., 2003). However, to date, most studies of termite mound abundance have relied only on observations of their natural abundance.

Termites are deeply embedded in savanna food webs and rely on dietary resources such as woody and herbaceous plant biomass that are strongly impacted by large mammalian herbivores. Browsing by cattle and wild ungulate herbivores can lead to changes in savanna grass, shrub, and tree density and diversity (Goheen et al., ,^2010^, ^2013^; Lagendijk et al., 2012; Odadi et al., 2011; Pringle et al., 2014; Veblen et al., 2016; Young et al., 2013). Elephants, in particular, are known to radically affect landscape heterogeneity by increasing the availability of downed woody debris in the short term (Holdo & McDowell, 2004; Kerley et al., 2008; Landman et al., 2019) and by reducing woody density and cover in the long term (De Beer et al., 2006; Laws, 1970; Whyte et al., 2003), and even modifying microclimates (Joseph et al., 2018). Herbivory by domestic and wild herbivores is also known to affect various other ecosystem traits, including herbaceous productivity (Charles et al., 2017) and nutrient cycling (Sitters et al., ,^2014^, ^2020^; Sitters & Olde Venterink, 2015). Recent studies have sought to understand how changes in herbivore communities may impact termites abundance and diversity (Freymann et al., 2010; Lagendijk et al., 2016). Although several studies have hypothesized feedback loops between mammalian herbivores and termites via changes in the availability of plant material (Freymann et al., 2010; Levick et al., 2010), few have tested these relationships experimentally (Lagendijk et al., 2016).

In this study, we examined how changes in savanna plant and herbivore community structure impacted the abundance of termite mounds. We used landscape‐scale variation in tree density and two long‐term experiments to measure the responses of termites to changes in resource abundance. We quantified termite mound abundance and areal cover a) along a gradient of tree density, b) in tree thinning plots, and c) in the Kenya Long‐term Exclosure Experiment (KLEE), a series of experimental plots that have excluded different combinations of elephants, other large wildlife, and cattle since 1995.

## 2 METHODS

### 2.1 Study Site

We conducted this research at Mpala Research Centre (0°17'N, 36°52'E) in central Kenya in a wooded grassland on “black cotton” vertisol soils. These high‐clay soils are characterized by shrink‐swell dynamics, impeded drainage, and relatively high nutrient content. Plant communities in this system are relatively species‐rich (>100 species), but dominated by five grass species that account for 85% of the herbaceous cover (see Appendix 1 in Porensky et al. 2013). The overstory of this wooded grassland ecosystem is dominated by *Acacia drepanolobium*, which accounts for 97% of woody cover (Young et al., 1998). The study area is host to both native and domestic mammalian ungulates, including domesticated cattle (*Bos taurus indicus*), plains zebra (*Equus quagga*), Grevy's zebra (*Equus grevyi*), African elephant (*Loxodonta africana*), giraffe (*Giraffa camelopardalis*), Grant's gazelle (*Gazella grantii*), African buffalo *(Syncerus caffer*), hartebeest (*Alcelaphus buselaphus*), eland (*Taurotragus oryx*), oryx (*Oryx beisa*), and steinbuck (*Raphicerus campestris*). For their relative abundances in this ecosystem based in aerial counts, see Table 1 in Veblen et al. (2016).

*Odontotermes* sp. termite mounds occur across this landscape (Pringle et al., 2010). *Odontotermes* mounds are slightly raised (<1 m) and have aboveground diameters ranging from <1 m–20 m. The larger, more conspicuous mounds are hyper‐dispersed (Pringle et al., 2010). In other species of fungus‐growing *Odontotermes* and *Macrotermes* termites, parameters including termite mound area have been shown to be strongly positively correlated with both termite colony population size and age (Collins, 1981; Darlington & Dransfield, 1987). Soils on mounds (in black cotton) are altered both chemically and physically, characterized by lower clay content and higher silt content (Darlington, 1985) and elevated soil and plant nutrient content (Brody et al., 2010). These termite mounds are generally treeless. In other ecosystems, trees on mounds may be preferred (Holdo & McDowell, 2004), or not (van der Plas et al., 2013), but the causes of treelessness of termite mounds in this ecosystem are not well understood.

### 2.2 Experimental manipulations (and a natural gradient)

We used two large‐scale manipulation experiments to test how termite mound densities responded to changes in large herbivore communities and two important termite dietary resources, woody biomass (here measured as tree density) and herbaceous aboveground net primary productivity (ANPP).

#### 2.2.1 Kenya Long‐term Exclosure Experiment (KLEE)

**FIGURE 1:**
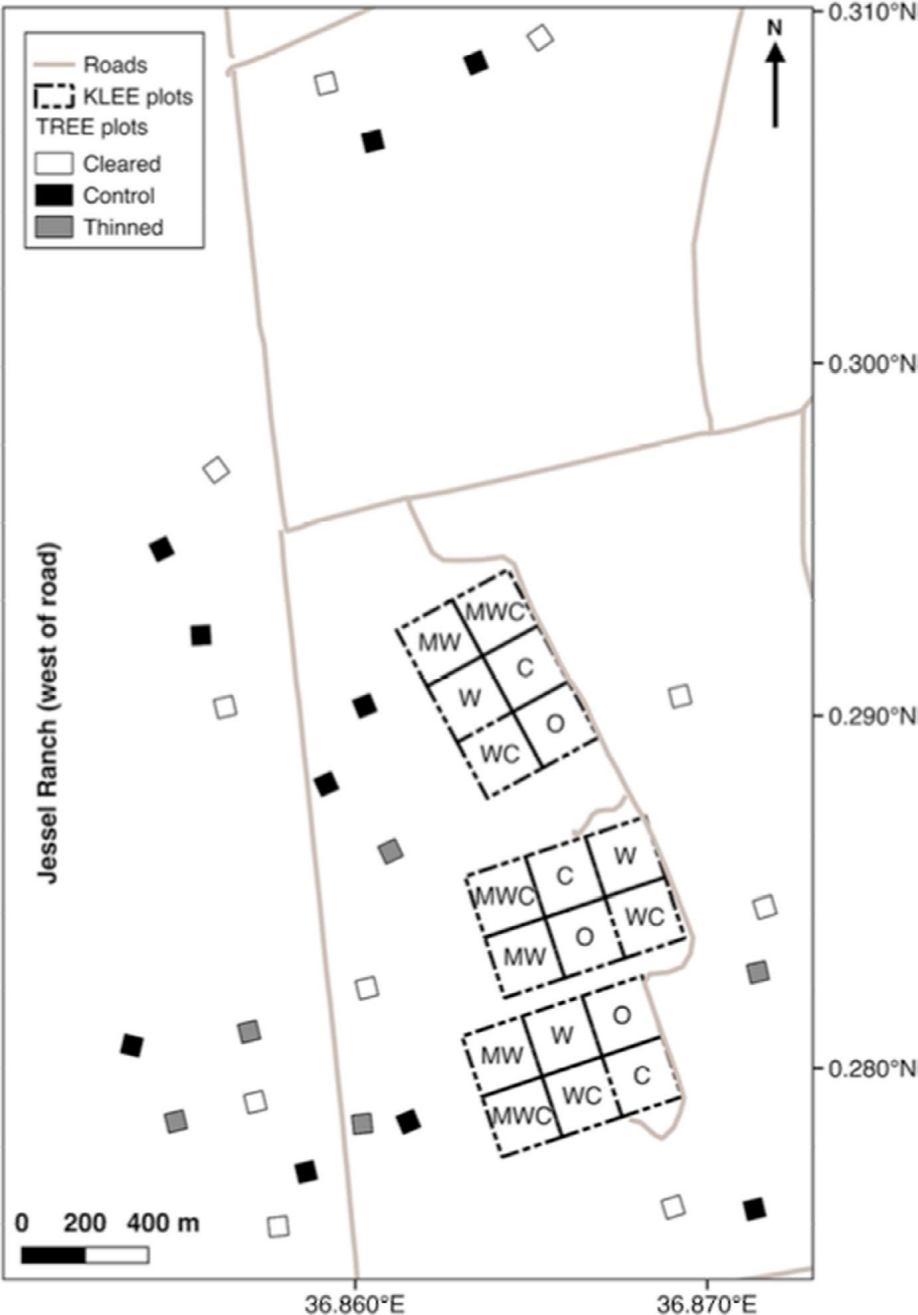
Map of the Kenya Long‐term Exclosure Experiment (KLEE) and the Tree Removal Ecosystem Experiment (TREE). In KLEE plots, letters represent the guilds of large herbivores present in each experimental plot. O = No herbivores, C = Cattle, W = Wildlife >15 kg, and *M* = Megaherbivores. TREE plots to the west of the North‐South boundary road are located on Jessel Ranch. All other plots are located on Mpala Research Center property

First, we surveyed abundance of termite mounds and termite dietary resources in the KLEE plots. KLEE was established in 1995 to examine how native and domestic large mammalian herbivores impact each other and their shared savanna ecosystem (Young et al., 1998). KLEE consists of eighteen 4‐hectare plots with semipermeable barriers that exclude different combinations of cattle (“C”), mesoherbivore wildlife >15 kg (“W”), and megaherbivores (elephants and giraffes; “M”). There are six treatments, each of which is replicated over three blocks: O, C, W, WC, MW, and MWC (Figure 1; for more details, see Young et al., 1998). The capital letter of each plot represent which herbivores are allowed access: “O” plots allow no herbivores >15 kg, “C” plots allow only cattle access, “W” plots allow only mesoherbivore wildlife access, “WC” plots allow access by both cattle and mesoherbivores, “MW” plots allow access by mesoherbivores and megaherbivores, and “MWC” plots allow access by mesoherbivores, megaherbivores, and cattle. In C, WC, and MWC plots, herded groups of 100–120 head of cattle are grazed 3–4 times per year, depending on forage availability. This grazing regime reflects a moderate stocking rate similar to the overall Mpala Ranch stocking rate (Odadi et al., 2007). Grazing rates are standardized across all KLEE plots.

We surveyed all termite mounds in KLEE in May 2014 and again in July 2015, scoring whether they were active or not. *Odontotermes* mounds in this ecosystem are generally treeless and tend to be dominated by the perennial bunchgrass *Pennisetum stramineum* (Pringle et al., 2010). Active termite mounds also feature vents for nest ventilation and humidity control (Pomeroy, 2005). In addition to topographical features, the edges of *Odontotermes* spp. termite mounds can be delineated from background vegetation by a visible shift in plant community composition. Combined, these features make mounds generally visible from a distance of well over ten meters, though small mounds can be hard to identify until in closer proximity. We conducted comprehensive searches for termite mounds by walking parallel 10 m‐wide transects covering the entire area of every 4‐ha KLEE plot. We excluded from our surveys termite‐rich anthropogenic glades that developed from long‐abandoned cattle corrals (see Veblen, 2012). For both surveys, we used a Trimble Juno 3B GPS to record the center and footprint of each mound.

To explore whether resource limitation is a driver of termite mound abundance, we quantified variation of two important termite dietary resources in the KLEE plots: tree density along a natural gradient across KLEE plots, and herbaceous productivity among KLEE treatments. KLEE plot tree densities were calculated from complete counts from lines transects of all trees ≥1 m tall in the central hectare of all 18 KLEE plots in January—March 2015. Tree density across KLEE blocks increases along a natural gradient from north to south. This tree density gradient occurs across an area with no measurable differences in precipitation, but significant differences in soil texture. These differences in soil texture correlate with differences in tree density (Riginos & Grace, 2008). Tree density in KLEE is strongly positively correlated with the abundance of fine woody debris (Kimuyu et al., 2014). We used a series of moveable productivity cages to quantify aboveground herbaceous net primary productivity in each of the 18 KLEE plots, quantifying the increase in biomass over 74‐month periods from June 2010 to September 2012 (and therefore robust against seasonal and interannual variation), in 1 m^2^ subplots protected from herbivory by small‐mesh cages (ANPP; see Charles et al., 2017 for details) and calculated plot‐level ANPP estimates by averaging measurements taken over ten collection periods from 2010 to 2012.

#### 2.2.2 Tree Removal Ecosystem Experiment (TREE)

As an experimental test of patterns from the natural gradient in tree density, in early 2006, and then again in May 2014, we also collected termite mound data from the Tree Removal Ecosystem Experiment (TREE), an experiment established in March 2006 (Riginos, 2014). A total of 25 plots are located on Mpala Research Center and directly adjacent Jessel Ranch (Figure 1). Plots are open and accessible to wild herbivores (similar to the MWC treatments in KLEE). While wildlife populations are comparable between these two ranches, cattle are stocked at higher rates on Jessel Ranch than on Mpala (C. Riginos, unpublished data). These experimental plots measure 60 × 60 m and have one of three treatments: control (*N* = 10), thinned tree densities (*N* = 5), and total tree removal (*N* = 10). In thinned plots, we removed a randomly selected set of two thirds of trees within each half‐meter size class (with trees ranging from <50 cm to >5 m) in order to maintain tree size class structure. These 25 plots are located along a natural north‐south gradient in tree density ~4 km in length, with premanipulation tree densities ranging from 268 to 2,784 trees ha^−1^ and post‐treatment tree densities ranging from 0 to 2,219 trees ha^−1^ (including controls). For additional details, see Riginos (2014). All tree removal plots are located within 2 km of KLEE on the same black cotton vertisols and are placed in locations with no signs of recent disturbance from fire or humans.

In TREE, we counted and mapped termite mound abundance in 2006 prior to tree removals, and then again in 2014 after 8 years of tree manipulation. For our 2006 mound survey, we recorded and hand‐mapped the locations of all termite mounds within each plot relative to a 10 × 10 m grid of tapes laid out in each plot. For our 2014 survey, we used a Trimble Juno 3B GPS with meter‐level accuracy to record the center and footprint of each mound.

### 2.3 Statistical analyses

All analyses were carried out in R version 3.3.2. We used linear mixed models (LMMs) to determine which factors were best predictors of termite dietary resource availability, individual termite mound size, and termite mound areal cover and abundance, including both active and inactive mounds. We used the nlme package (Pinheiro et al., 2015). We checked the normality and homoscedasticity of the residuals of all models to assure that they met assumptions. We used Tukey's HSD analyses for post hoc comparisons of different herbivore treatments. We used the MuMIn package (Bartón, 2018) to calculate the marginal and conditional *R* ^2^ values for our models (i.e., the variance explained by only fixed effects and the variance explained by both fixed and random effects, respectively).

In KLEE, we first asked how termite mound abundance, areal cover, and individual mound size varied between herbivore exclusion plots in 2014 and 2015. We created LMMs with termite mound abundance and areal cover as response variables and in a 2 × 3 design that included all six KLEE treatments, the effects of cattle (2 levels: present or absent; O, W, and MW plots vs. C, WC, and MWC plots), and wildlife (3 levels: no wildlife [O and C plots], mesoherbivores only [W and WC plots], or megaherbivores and mesoherbivores [MW and MWC plots]) as predictor variables. Block was included as a random effect. We used a compound symmetry covariance structure to address the nonindependence of the 2 years of termite mound surveys within the same plots. Because we were interested in potential differences in termite colony age and population size between herbivore treatments, we also asked if herbivores affected the aboveground size of individual termite mounds, a proxy for both age and population size (Darlington & Dransfield, 1987; Josens & Soki, 2010). We created a hierarchical LMM to test the effects of cattle and wildlife on the size of individual termite mound areas. Block and plot nested within block were treated as random variables. We again used a compound symmetry covariance structure to address the nonindependence of the 2 years of termite mound surveys within the same plots.

In order to test the potential indirect feedbacks and mechanisms between large herbivores and termite mound abundance, we next asked whether large herbivores influenced the abundance of an important termite dietary resource, woody biomass (here measured as tree density). To model the effects of large herbivores on tree density, we created a LMM with cattle presence (2 levels: present or absent), mesoherbivore presence (2 levels: present or absent), and megaherbivore presence (2 levels: present or absent) as predictor variables. We included mesoherbivores and megaherbivores as separate predictor variables in this model because we were particularly interested in the quantifying the effects of megaherbivores on plot tree density. We also included the Northing centroid of each plot as a predictor variable to test and control for the previously reported underlying N‐S tree density gradient (Riginos & Grace, 2008). Tree density from the central hectare of each KLEE plot was included as a response variable. Previous work in this system has already shown that ANPP and herbivore grazing intensity are strongly positively correlated (Charles et al. 2016). Grazing pressure is greatest in plots where cattle are present and generally increases with the addition of each herbivore guild (i.e., grazing intensity in MWC > WC >C > MW >W > O; see Veblen et al. 2016).

Finally, we created a “termite dietary resources” linear mixed model to test whether termite dietary resources affected termite mound abundance or areal cover. Our termite dietary resources models included termite mound abundance and areal cover as our response variables, and tree density and herbaceous ANPP as predictor variables. We included block as a random effect and used a compound symmetry covariance structure to address the nonindependence of the 2 years of mound surveys within the same plots.

In TREE, we asked how termite mound abundance across the 60 × 60 m treatment plots varied with baseline tree density and responded to changes in tree density. Because plots were located on two directly adjacent ranches with different cattle densities, we included ranch identity as a random effect in all models. We first created a LMM that included baseline tree density as a predictor variable and 2006 termite mound abundance as a response variable. To test whether tree removal treatments predicted termite mound abundance after 8 years, we created a LMM with plot post‐treatment tree density and the surround baseline tree density as predictor variables and 2014 mound abundance as a response variable. Ranch was included as a random effect.

## 3 RESULTS

### 3.1 Termite mound patterns in KLEE

**FIGURE 2:**
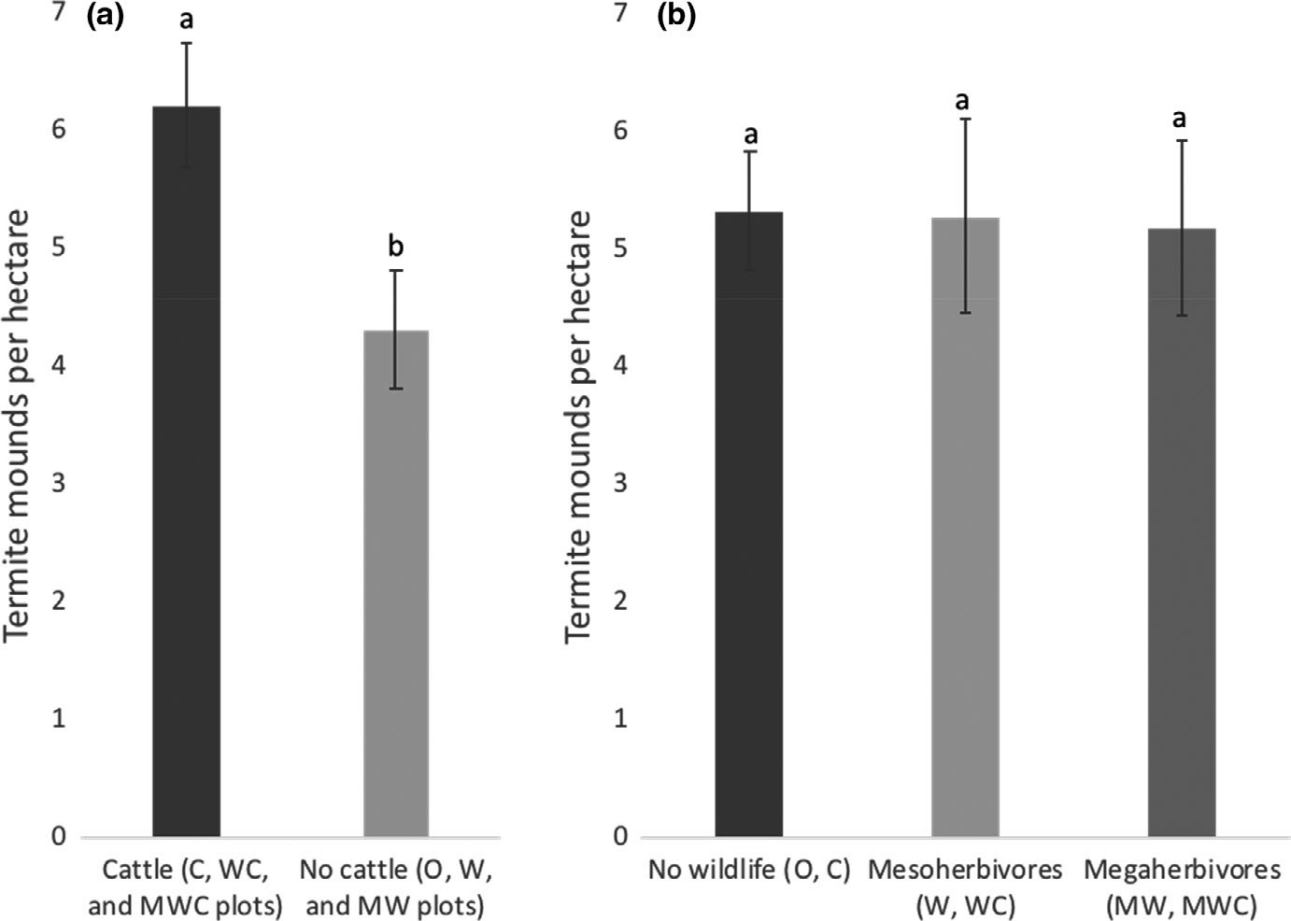
Herbivore effects by guild on termite mound abundance per hectare in the KLEE plots. Error bars represent one standard error. Termite mound abundance was significantly affected by the presence of cattle, but not mesoherbivores or megaherbivores. For each graph, significant differences (*p* < 0.05, Tukey's HSD) among herbivore treatments are indicated with letters. (a) Termite mound abundance in noncattle (O, W, and MW) versus cattle (C, WC, and MWC) plots. (b) Termite mound abundance in nonwildlife plots (O and C) versus mesoherbivore wildlife (W and WC) and megaherbivore wildlife (MW and MWC) plots

**FIGURE 3:**
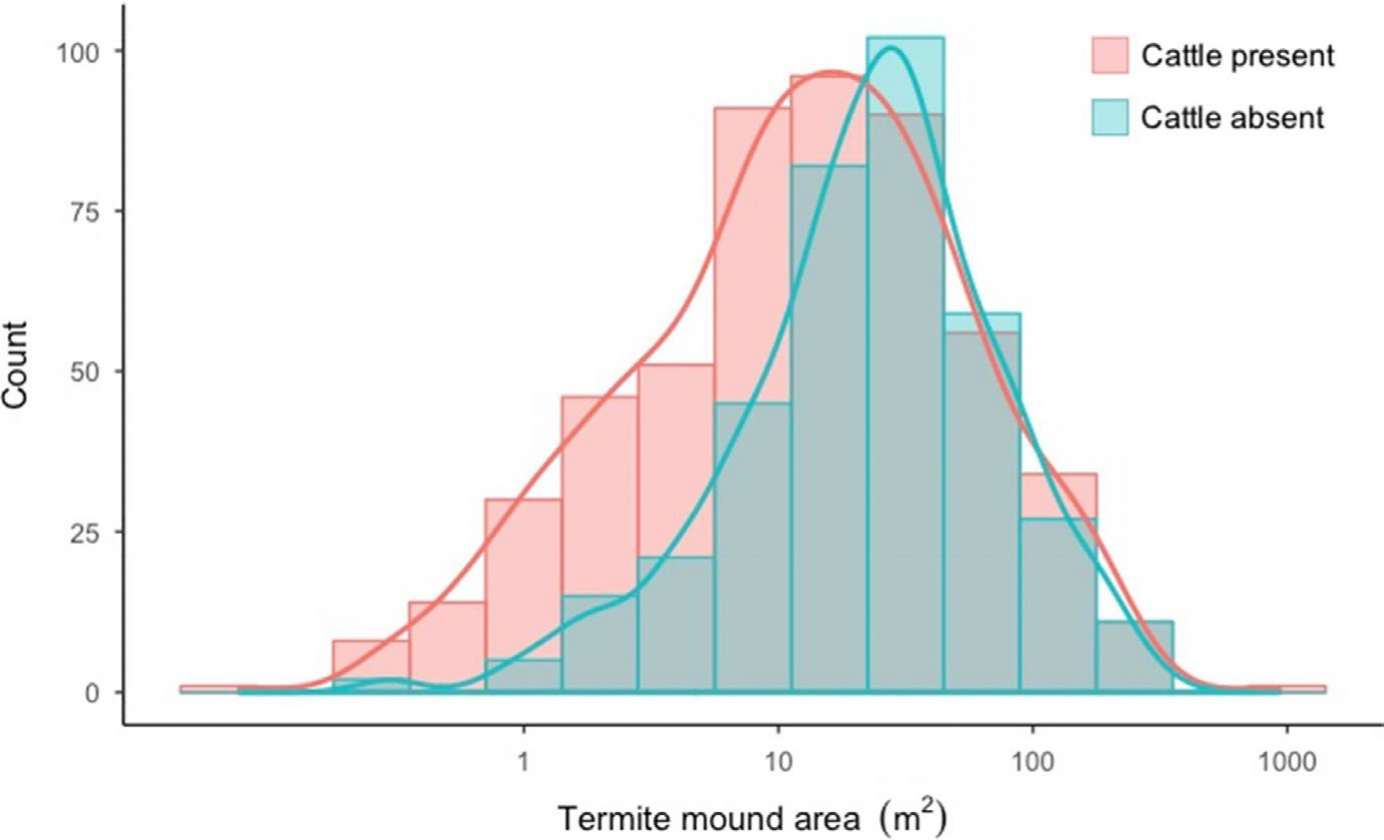
Histogram of termite mound size in KLEE plots where cattle are present (C, WC, and MWC) or absent (O, W, and MW). Corresponding lines represent the kernel density estimate for each distribution

In KLEE, termite mound densities ranged from 2.75 to 8.25 mounds ha^−1^, and their footprints comprised approximately 1%–3% of the area of the experimental plots. Termite mound abundance was significantly affected by the presence of cattle; after accounting for differences in tree density, mounds were approximately 60% more abundant in plots with cattle than in those without (C, WC, and MWC plots vs. O, W, and MW plots; *F*_1,29_ = 8.62, *p* = 0.006, c*R* ^2^ = 0.43; Table 1; Figure 2). Although termite mound areal cover was also greater in cattle plots, this difference was not significant (*F*_1,29_ = 1.26; *p* = 0.27; Table 1). The presence of wildlife guilds (i.e., O and C plots with no wildlife; W and WC plots with mesoherbivore wildlife; and MW and MWC plots with both mesoherbivore and megaherbivore) were not significant predictors of termite mound abundance (*F*_2,29_ = 0.02; *p* = 0.86; Table 1) or areal cover (*F*_2,29_ = 0.39, *p* = 0.68; Table 1). It appears that the increased abundance of termite mounds in the cattle treatments of KLEE was due to an increased number of smaller mounds. The number of larger mounds was essentially unchanged between cattle and noncattle plots (Figure 3). The mean size of termite mounds in cattle plots was 56% less on average than plots without cattle (*F*_1,13_ = 14.65, *p* = 0.002; Table 1). Termite mound size was not significantly affected by mesoherbivore or megaherbivore wildlife treatments (*F*_2,13_ = 0.59, *p *= 0.57; Table 1).

**TABLE 1:** Linear mixed model results for the relationships between herbivore treatments, termite dietary resources, and termite mound abundance, areal cover, and size. Random effects for each model are specified in the methods section. Bolded results are significant at the *p* = 0.05 level

Model	Response Variable	Predictor Variable (fixed)	*df*	*F*
Effects of herbivores on plot termite mound abundance	Termite mound abundance	**Cattle Presence**	**1**	**8.62**
		Wildlife guild presence	2	0.02
Effects of herbivores on plot termite mound areal cover	Termite mound areal cover	Cattle presence	1	1.26
		Wildlife guild presence	2	0.39
Effects of herbivores on individual termite mound size	Individual termite mound size	**Cattle presence**	**1**	**14.7**
		Wildlife guild presence	2	0.59
Effects of herbivores on termite dietary resources (tree density)	Tree density	Cattle Presence	1	0.13
		Mesoherbivore presence	1	0.49
		**Megaherbivore presence**	**1**	**5.34**
Effect of termite dietary resources on termite mound abundance	Termite mound abundance	**Herbaceous ANPP**	**1**	**5.79**
		**Tree density**	**1**	**5.89**
Effect of termite dietary resources on termite areal cover	Termite mound areal cover	Herbaceous ANPP	1	0.02
		Tree density	1	2.77
Effect of baseline tree density on termite mound abundance	Baseline termite mound abundance	**Baseline tree density**	**1**	**7.81**
Effect of tree density treatments on termite mound abundance	Post‐treatment termite mound abundance	**Post‐treatment tree density**	**1**	**12**
		**Background tree density**	**1**	**7.89**

Large herbivores significantly impacted the availability of termite dietary resources. By 2015, plots in KLEE which allowed megaherbivores contained 18% fewer trees across all blocks than those that excluded megaherbivores (*F*_1,11_ = 5.34, *p* = 0.04; Table 1). In contrast, neither cattle (*F*_1,11_ = 0.13, *p* = 0.72; Table 1) nor mesoherbivores (*F*_1,11_ = 0.49, *p* = 0.48; Table 1) were significant drivers of tree density. In addition, previous work in these plots has already shown a strong positive correlation between grazing intensity and herbaceous ANPP (Charles et al. 2016).

**FIGURE 4:**
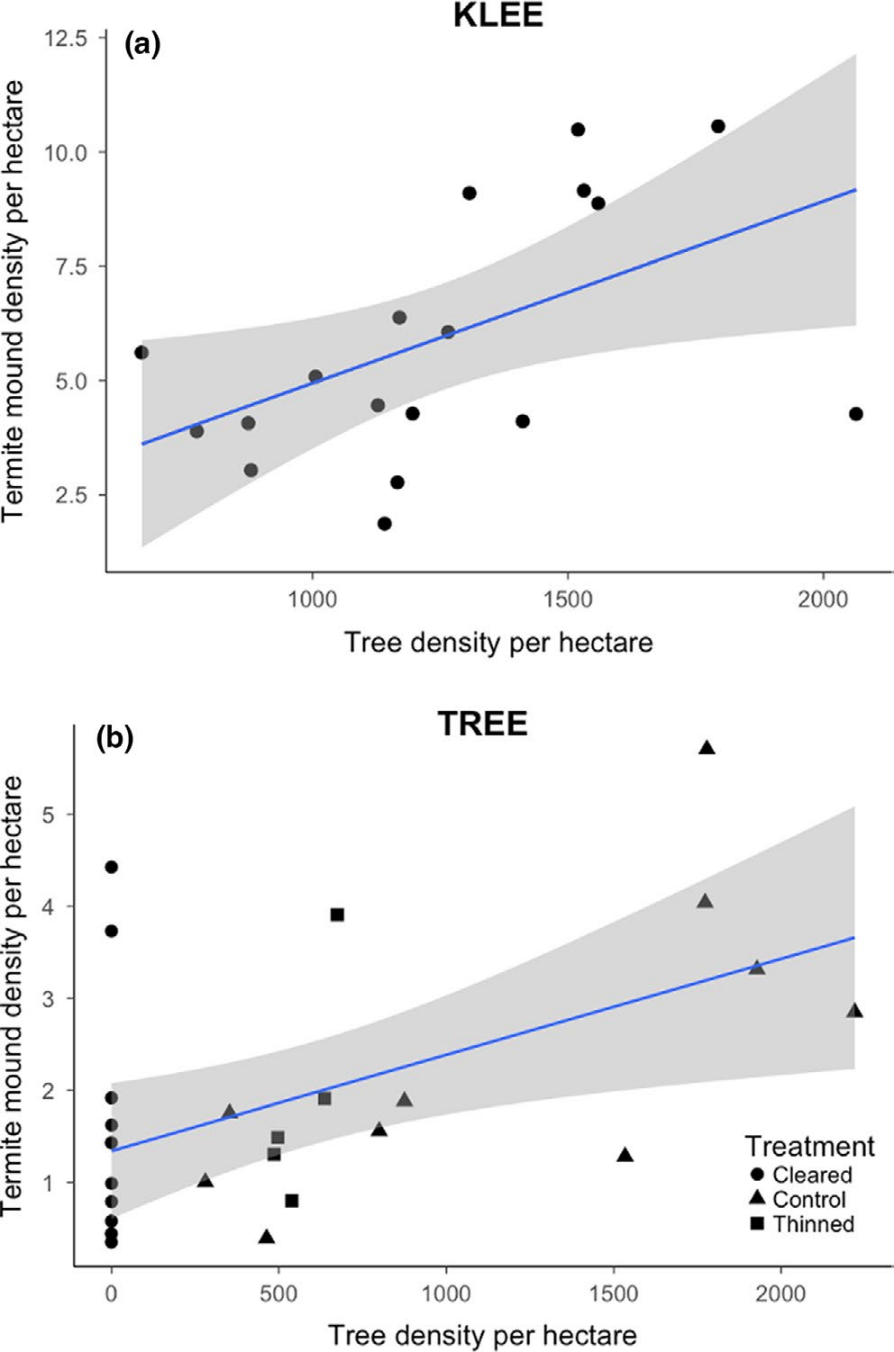
Relationships between current tree density and termite mound abundance per hectare in (a) TREE plots after 8 years of treatment and (b) KLEE plots after 20 years of treatment. Dotted lines represent fitted linear relationships between variables. Tree density was a significant predictor of termite mound abundance in both experiments

We modeled the effects of termite dietary resource availability on termite mound abundance and areal cover. Termite mound density was positively correlated with tree density along the natural N‐S gradient across KLEE (*F*_1,30_ = 5.89, *p* = 0.02; Figure 4; Table 1). Termite density was also positively correlated with herbaceous ANPP among KLEE treatments (*F*_1,30_ = 5.79, *p* = 0.02). In contrast, termite mound areal cover was only marginally positively correlated with tree density (*F*_1,30_ = 2.77, *p* = 0.11), and not significantly correlated with herbaceous ANPP (*F*_1,30_ = 0.02, *p* = 0.88; Table 1).

### 3.2 Termite mound patterns in TREE

In the tree removal plots, mound abundance in 2014 ranged from 0 to 16.7 mound ha^−1^. After controlling for differences in tree density, plots located on Jessel Ranch, which has higher cattle presence in our plots, had twice the density of termite mounds than the plots located on Mpala, which has had less cattle presence in the TREE plots since 2008 (*F*_1,21_ = 10.14, *p* = 0.004; Table 1). Our initial (baseline) 2006 survey of termite mound abundance showed that plots with the highest baseline tree densities had the highest initial termite mound abundances (*F*_1,22_ = 7.81, *p* = 0.01; Table 1). This accords with the positive effect of tree density on mound abundance we observed in KLEE. After controlling for this, we also found evidence for the impact of experimental tree removal on termite mound abundance. Post‐treatment mound density was strongly positively correlated with post‐treatment tree density (*F*_1,21_ = 12.02, *p* = 0.002; Figure 4b; Table 1) and surrounding baseline tree density (*F*_1,21_ = 7.89, *p* = 0.01).

## 4 DISCUSSION

**FIGURE 5:**
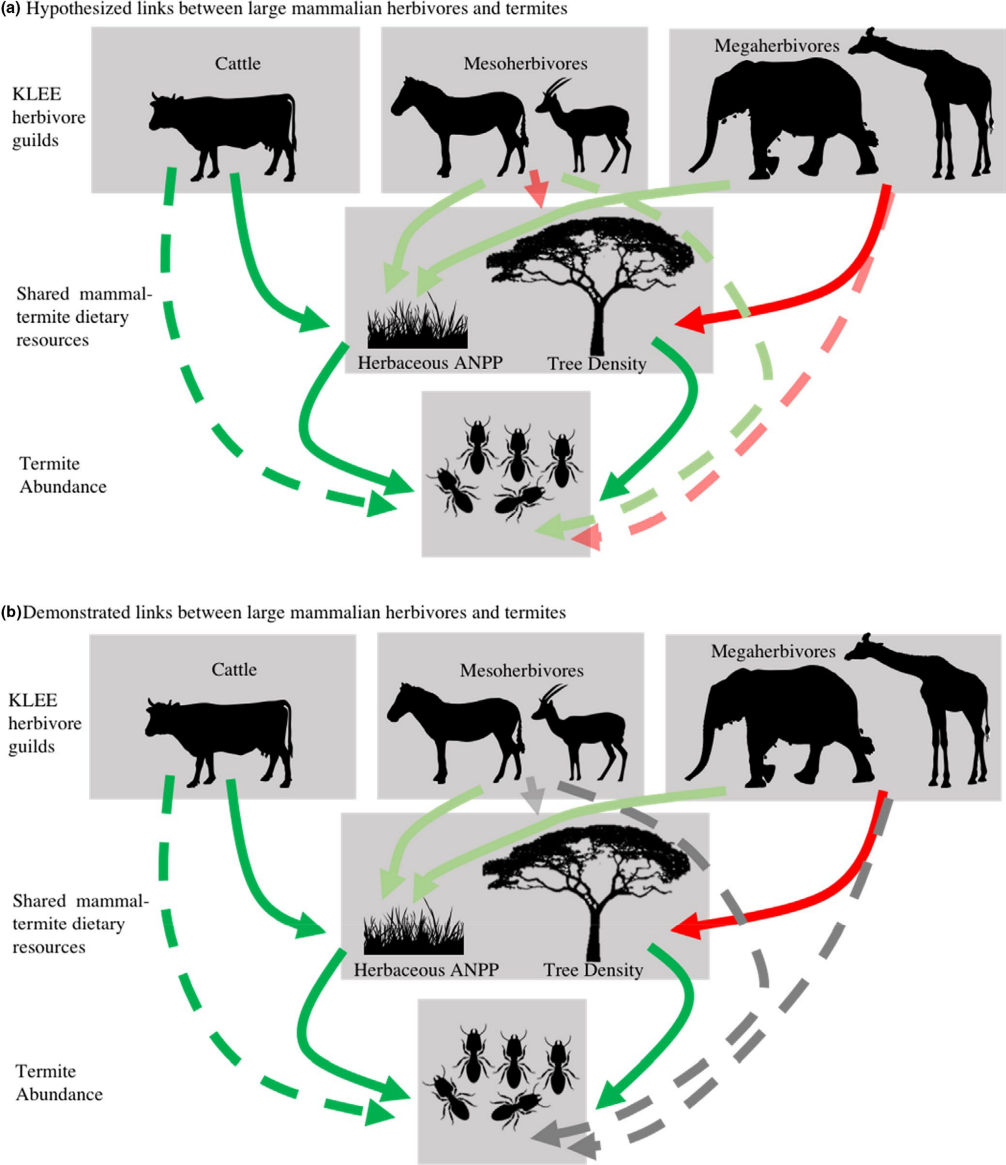
Hypothesized and demonstrated relationships between KLEE herbivore treatments, shared termite‐herbivore dietary resources, and termite mound abundance. Solid lines indicate direct relationships while dotted lines indicate assumed indirect relationships between variables. Red lines indicate a negative relationship between variables, green lines indicate a positive relationship between variables, and gray lines indicate a nonsignificant relationship between variables at the *p* = 0.05 level. For the relationship between different herbivore guilds and herbaceous ANPP, the bolder green indicates a stronger relationship between cattle and herbaceous ANPP than either mesoherbivore wildlife or megaherbivore wildlife (data taken from Charles et al., 2017). “Cattle” refers to the comparison between KLEE plots where cattle are present (C, WC, and MWC) and plots where cattle are not present (O, W, and MW). Effects of mesoherbivores (W and WC plots) and megaherbivores (MW and MWC plots) were compared to each other and to KLEE plots without wildlife (O and C plots)

Our study provides unique descriptive and experimental evidence linking termite mound abundance to the availability of dietary resources. Further, we showed how two ecosystem engineers in this system, large mammalian herbivores and termites, are linked in previously unexplored ways (Figure 5). Fungus‐cultivating termites like those in our study are known to consume broad diets that include animal byproducts as well as woody and herbaceous plant material (Freymann et al., ,^2007^, ^2008^; Schuurman, 2006). We demonstrate that changes in these termite dietary resources appear to drive and alter patterns in termite mound abundance, and that these resources can be modulated by the presence of large herbivores (cattle, directly, and elephants by inference). These findings suggest that large herbivores may be influencing termite abundance in ways that are simultaneously positive (e.g., stimulating herbaceous productivity) and negative (e.g., reductions in tree density), and that these effects can vary by herbivore guild. Our results also suggest that patterns of termite mound abundance are more dynamic than previously thought (Darlington, 1985; Pomeroy, 2005; Pringle & Tarnita, 2017). Understanding and quantifying the diverse array of direct and indirect interactions between organisms will broaden our understanding of savanna networks.

### 4.1 Termite response to resource availability

Results from our herbivore exclusion experiment and tree removal experiment both tell a similar story: termite mound abundance (and, marginally, areal cover) is influenced by the availability of termite dietary resources. In KLEE, termite mound abundance was positively correlated with tree density and herbaceous ANPP. Meanwhile, termite mound areal cover was marginally positively correlated with tree density, but not herbaceous ANPP. The apparent explanation for the differences in our findings between termite mound abundance versus areal cover is the turnover rates of small colonies versus large colonies. Differences in mound abundance between KLEE plots are driven largely by the presence of smaller‐sized mounds in plots that allowed cattle (Figure 3). Work in other systems has shown that smaller termite mounds tend to be occupied by newer colonies and experience higher turnover rates than larger mounds (Collins, 1981). It is therefore not surprising that the effects of biotic drivers on mound areal cover were less than their effects on mound abundance.

We did not quantify the effects of an additional potential dietary resource, herbivore dung. Because herbaceous ANPP is strongly positively correlated with herbivore grazing intensity (Charles et al. 2016) and plot dung density, we were not able to disentangle the relative importance of herbaceous ANPP versus herbivore dung for termite diets. Previous studies have identified both dung and grass litter as important termite resources (Freymann et al., 2008; Korb & Linsenmair, 2001), so it is possible that both resources positively contribute to overall termite abundance in this system.

Tree density was an important driver of termite mound abundance in both KLEE and TREE. We found a strong positive association between termite mound abundance and tree density along a natural gradient across the KLEE and TREE blocks (Figure 4). More powerfully, experimental data from TREE demonstrated a direct positive link between tree density and termite mound abundance. Together, these results provide evidence that termite colonies track changes in woody biomass availability, and that significant changes in mound abundance can occur over relatively short time periods (8 years in the case of TREE, and 20 years in the case of KLEE). While other studies have linked termite mound abundance and tree density (Davies et al., 2014), none have previously demonstrated this link experimentally. Results from TREE are also the first to demonstrate that termite colonies dynamically respond to changes in resource availability over relatively short time periods (8 years).

Our finding that tree density drives patterns in termite abundance is not surprising in the context of other functionally similar termite species. Many species of termites in the genus *Odontotermes* and the functionally comparable *Macrotermes* subsist on diets dominated by dead woody biomass (Brune, 2014; Donovan et al., 2001). Plots with higher tree densities have more dead woody debris on the ground (Kimuyu et al., 2014). In addition, the amount of tree damage may be magnified in high tree density areas since elephants appear to browse more in these areas than areas with fewer trees (Riginos & Grace, 2008) and typically pull down more woody biomass than they consume (Lagendijk et al., 2016).

In both KLEE and TREE, there were strong pre‐existing gradients in tree density. The biotic and abiotic conditions underlying this variation in tree density might also be important for our understanding of termite mound spatial dynamics. This tree density gradient appears to be related to small differences in soil texture (Riginos & Grace, 2008). It is also possible that differences in soil could influence termite mound densities (hence, the importance of our experimental approach). Soil and habitat type have been suggested as drivers of patterns in termite mound abundance in other systems (Davies et al., 2014; Korb & Linsenmair, 2001). While it is possible that these soil differences could also influence termite mound abundance in ways we did not explore, results from the tree thinning experiment isolate tree density itself as a major driver of termite mound density, which is why our block effects were so strong, given the N–S gradient in tree density in KLEE.

### 4.2 Herbivores and termite resource availability

Our exclosure experiment revealed a range of pathways through which herbivores influence termite resource abundance. Importantly, we showed that termite mound abundance was linked to dietary resource availability, and that this resource availability could be altered by the presence of large herbivores. The influence of herbivores on termite dietary resources varied by guild and was not uniformly positive or negative. While megaherbivores had a significant negative impact on plot tree density, mesoherbivores, and cattle did not. Meanwhile, herbaceous ANPP is strongly positively correlated with both overall grazing intensity and the presence of cattle, but not the presence of mesoherbivore or megaherbivore wildlife (Charles et al. 2016).

Overall, the abundance of termite mounds was strongly positively linked to the presence of cattle, but not significantly linked to the presence of mesoherbivore or megaherbivore wildlife. There are at least two pathways by which cattle may influence termite abundance, both due to the influence of herbivores on termite resource availability. First, at the stocking rates in this site, cattle grazing stimulates aboveground herbaceous productivity (Charles et al. 2016), a termite food source (Boutton et al., 1983; Freymann et al., 2007; Schuurman, 2006). Second, cattle (in higher numbers than wildlife; see Table 1 in Veblen et al. 2016) deposit considerable amounts of nitrogen‐rich dung, another potential termite dietary resource. Unlike megaherbivores, cattle did not significantly influence tree density.

We did not find significant experimental effects of the presence of mesoherbivores or megaherbivores on termite mound abundance. While this is surprising in light of the strong positive effects that cattle have on mound abundance and size, it is possible that mesoherbivores and megaherbivores are impacting termite populations and resource availability in both positive and negative ways that at least partly offset each other. In contrast to cattle, wild herbivores tend to homogenize patterns in aboveground productivity in space and time (Charles et al. 2016); this homogenization may make termite resource acquisition and colony abundance more stable through time. Like cattle, mesoherbivores and megaherbivores also deposit significant amounts of dung, another potential termite dietary resource.

Megaherbivores reduced tree densities by 18% across KLEE plots. However, while megaherbivore browsing reduced tree density, it can also simultaneously (and temporarily) increase tree damage and the availability of dead woody biomass (Holdo & Mcdowell, 2004; Kerley et al., 2008; Pringle, 2008). Therefore, (a) there may have been as yet insufficient net effect of the reduction of tree density (18%) by megaherbivores on woody biomass availability to negatively impact termite populations, or (b) it was being offset by their positive effects in damaged woody debris.

Differences in abundance of termite mounds across herbivore treatments appeared to be driven by the formation of small termite mounds. This result may explain why the presence of cattle was a significant predictor of termite mound abundance, but not total areal cover. In plots where cattle graze, smaller mounds are more abundant, whereas larger mounds are not. Small termite mounds like those found in plots with cattle are likely to be younger colonies (Collins, 1981; Korb & Linsenmair, 2001). The fact that few small termite mounds are found in noncattle plots (Figure 3) suggests that there are fewer new colonies being formed in these plots. The formation of new colonies may be in response to pulses of available resources such as aboveground herbaceous cover or dung. Cattle are grazed in the KLEE plots episodically (as they have been traditionally throughout East Africa), providing pulses of dung and vegetative growth during those times. Previous work on the arrangement and density of termite mounds has focused on the regular distribution of larger mounds (Davies et al., 2014; Pringle et al., 2010), although there is some evidence for smaller termite mounds being distributed less regularly than larger mounds (Grohmann et al., 2010, Muvengwi et al., 2018). Understanding the dynamics of both large and small mounds may give us a better understanding of termite colony dynamics and provide insight into our findings.

### 4.3 Positive and negative feedbacks between tree density and termite mound abundance

There is another feedback among these ecosystem engineers in need of further study. While overall termite mound abundance appears to be largely driven by woody biomass, *Odontotermes* mounds themselves are treeless. This may limit the total number of termite mounds in a landscape by reducing overall tree density and the productivity of existing mounds. However, it is not clear how termite mounds affect overall tree abundance in the landscape. These aspects of mound footprints comprise only 1%–3% of the landscape in this system, so these feedback pathways may be relatively small, but nonetheless impactful.

## 5 CONCLUSIONS

We have demonstrated complex interactions among two types of powerful savanna ecosystem engineers: termites and large mammalian herbivores. These interactions are likely to alter larger‐scale patterns of ecosystem structure and function. Our understanding of ecosystems and their engineers should not only include main effects, but also their interactions, which are likely to often be cryptic and best revealed by landscape‐scale multifactorial experiments such as those reported here. A particularly important takeaway of these surveys was the diversity of pathways through which termites and large herbivores interact. These pathways appear to have both positive and negative effects on termite mound abundance. Understanding how ecosystem engineers control the flow and availability of resources gives us a richer understanding of species interactions in savanna ecosystems.

## CONFLICT OF INTEREST

None declared.

## AUTHOR CONTRIBUTION

**Grace K. Charles:** Conceptualization (equal); Formal analysis (equal); Investigation (equal); Writing‐original draft (lead). **Corinna Riginos:** Conceptualization (equal); Methodology (equal); Supervision (equal); Writing‐review & editing (equal). **Kari Veblen:** Conceptualization (equal); Project administration (equal); Supervision (equal); Writing‐review & editing (equal). **Duncan Kimuyu:** Project administration (equal); Supervision; Writing‐review & editing. **Truman P Young:** Conceptualization (equal); Project administration (equal); Supervision (equal); Writing‐review & editing (equal).

## Supporting information

Table S1

## Data Availability

Charles, Grace (2021): Data to accompany "Termite mound cover and abundance respond to herbivore‐mediated biotic changes in an African savanna". figshare. Dataset. https://doi.org/10.6084/m9.figshare.13557902.v1
