# Supplementary material for: Termite mound cover and abundance respond to herbivore‐mediated biotic changes in a Kenyan savanna
Source: Ecol Evol. 2021 Jun 3;11(12):7226–38. doi: 10.1002/ece3.7445 (PMC8216887; doi:10.1002/ece3.7445)
Supplement: Supplementary file 1 — Table S1 [file ECE3-11-7226-s001.docx]

Table S1. Geographic location (northing), Tree density (per ha), and NPP estimates for the 18 KLEE plots. Number of mounds/ha (excluding glades) were averaged over two counts (2014 and 2015). NPP Values from Charles et al. 2017.

| **Block** | **Treatment** | **GPS N** | **Trees >1m** | **NPP** | **Standardized NPP** | **Mounds per ha** |
| --- | --- | --- | --- | --- | --- | --- |
| C | O | 31365 | 853 | 5.21 | 0.44 | 4.27 |
| N | O | 32040 | 698 | -11.15 | 0.07 | 5.36 |
| S | O | 30983 | 1515 | 3.81 | 0.40 | 4.17 |
| C | W | 31622 | 854 | -5.23 | 0.20 | 2.26 |
| N | W | 32117 | 570 | -14.05 | 0.00 | 2.88 |
| S | W | 30793 | 1056 | 4.31 | 0.42 | 4.43 |
| C | MW | 31299 | 840 | -5.07 | 0.20 | 3.00 |
| N | MW | 32295 | 575 | 9.73 | 0.54 | 3.13 |
| S | MW | 31050 | 1063 | 7.62 | 0.49 | 9.32 |
| C | C | 31552 | 701 | 22.91 | 0.84 | 4.26 |
| N | C | 32216 | 746 | 29.07 | 0.98 | 5.38 |
| S | C | 30929 | 1052 | -0.89 | 0.30 | 8.55 |
| C | WC | 31435 | 998 | 24.83 | 0.88 | 9.05 |
| N | WC | 31942 | 537 | 20.59 | 0.79 | 4.67 |
| S | WC | 31119 | 1205 | 30.04 | 1.00 | 8.40 |
| C | MWC | 31486 | 590 | 9.82 | 0.54 | 3.88 |
| N | MWC | 32393 | 408 | 17.68 | 0.72 | 4.75 |
| S | MWC | 30862 | 966 | 3.18 | 0.39 | 7.05 |
